# CD19-negative relapse of pediatric B-cell precursor acute lymphoblastic leukemia following blinatumomab treatment

**DOI:** 10.1038/s41408-017-0023-x

**Published:** 2017-12-20

**Authors:** E. Mejstríková, O. Hrusak, M. J. Borowitz, J. A. Whitlock, B. Brethon, T. M. Trippett, G. Zugmaier, L. Gore, A. von Stackelberg, F. Locatelli

**Affiliations:** 1 Department of Pediatric Hematology/Oncology, CLIP, Charles University, Prague, Czech Republic; 2 Hematologic Pathology, Johns Hopkins University, Baltimore, MD USA; 3 University of Toronto, Hospital for Sick Children, Toronto, ON Canada; 4 Department of Pediatric Hematology, Robert Debre Hospital, Paris, France; 5 Pediatric Oncology, Memorial Sloan Kettering Cancer Center, New York, NY USA; 6 Global Development, Amgen Research (Munich) GmbH, Munich, Germany; 7 University of Colorado School of Medicine and Children’s Hospital Colorado, Aurora, CO USA; 8 Charité University Medical Center, Berlin, Germany; 9 Department of Pediatric Hematology-Oncology, IRCCS Ospedale Bambino Gesù, Rome, Italy; 10 University of Pavia, Pavia, Italy

CD19-negative relapse in B-cell precursor acute lymphoblastic leukemia (ALL) is observed as an infrequent event after chemotherapy and in up to 20% of patients after CD19-directed chimeric antigen receptor (CAR) T-cell immunotherapy^1^. Patients with CD19-negative relapse usually have a poor prognosis^1, 2^. The mechanisms underlying CD19-negative relapse are not fully understood but are important to elucidate to further optimize CD19-directed immunotherapies^3, 4^. Monitoring blasts in patients with CD19-negative relapse by flow cytometry is challenging due to the lack of cell surface markers other than CD19 that are consistently expressed. Furthermore, CD19 is often used as a parameter to quantify minimal residual disease (MRD) and diagnose relapse. Potential markers to monitor persistent or recurrent leukemic blasts in an emergent CD19-negative blast population include B-cell lineage antigens (CD20, CD22, CD24, and intracellular [i]CD79a) and the common ALL antigen CD10^5^.

Blinatumomab is an anti-CD3/CD19 bispecific T-cell engager (BiTE^®^) antibody construct indicated to treat patients with relapsed/refractory B-precursor ALL^6–8^. In a phase 1/2 multicenter trial, blinatumomab monotherapy showed 39% (*n* = 27) complete remission in 70 pediatric patients with relapsed/refractory ALL, with 14 patients achieving complete MRD response^9^. Seventy-one percent of patients had experienced relapse within 6 months after last treatment, demonstrating an unfavorable prognosis. At the end of a 2-year follow-up, 19 total patients relapsed (2 were still alive at the last assessment, 15 died, and 2 withdrew consent)^9^. Here, we present four pediatric patients (two each from phase 1 and 2) who experienced CD19-negative relapse and one patient with CD19-negative progression during treatment.

Detailed descriptions of study design, patient eligibility, dose modifications, interruptions, and discontinuations were previously reported^9^ and flow cytometry and MRD analyses (flow cytometry and polymerase chain reaction (PCR)) are summarized in the Supplementary Methods.

Briefly, a panel of 29 (patients #1–4) or 20 (patient #5) markers were used for flow cytometry analysis, as detailed in the Supplementary Methods. Samples were measured on cytometers (BD^TM^ LSR II (BD Biosciences, San Jose, CA, USA) or Dako CyAn^TM^ (DakoCytomation, Glostrup, Denmark)), and analyses were performed using the FlowJo^®^ software, version 8.5.3 or 7 (FlowJo, LLC, Ashland, CA, USA).

The study completion date was 24 May 2016. Baseline characteristics of patients with either CD19-positive or CD19-negative relapse (Table 1) were consistent with those of the entire patient population^9^. Flow cytometric profiles at study entry and relapse are summarized in Supplementary Table 1.

**Table 1:** Demographic and baseline characteristics of patients with CD19-positive or CD19-negative relapse^a^

	CD19-positive relapse (*N* = 14)	CD19-negative relapse (*N* = 4)
Sex, *n* (%)		
Male	10 (71)	2 (50)
Female	4 (29)	2 (50)
Geographic region, *n* (%)		
European Union	6 (43)	4 (100)
United States	8 (57)	0 (0)
Age, median (range), years	6 (1–17)	8 (5–12)
Age group,* n* (%)		
<2 years	3 (21)	0
2–6 years	6 (43)	2 (50)
7–17 years	5 (36)	2 (50)
Genetic abnormalities, *n* (%)		
* MLL* total	9 (64)	1 (25)
* MLL*-AF4 t(4, 11)	2 (14)	0
Other *MLL*	6 (43)	1 (25)
* BCR-ABL*	0 (0)	0 (0)
Hypodiploidy	1 (7)	0 (0)
Constitutional trisomy 21	1 (7)	0 (0)
Previous alloHSCT, *n* (%)		
Yes	9 (64)	1 (25)
No	5 (36)	3 (75)
Previous relapses, *n* (%)		
1	4 (29)	1 (25)
2	8 (57)	2 (50)
≥3	2 (14)	1 (25)
Refractory disease, *n* (%)	14 (100)	4 (100)
Yes	0 (0)	0 (0)
No	14 (100)	4 (100)
Time between last relapse and first blinatumomab infusion, median (range), months	0.9 (0.1–10.2)	0.8 (0.3–2.3)
Relapse within 6 months after last prior treatment attempt, *n* (%)	4 (29)	2 (50)
Bone marrow blast count (central laboratory), *n* (%)		
<50%	6 (43)	1 (25)
≥50%	8 (57)	3 (75)

*alloHSCT* allogeneic hematopoietic stem cell transplantation; *BCR-ABL* breakpoint cluster region-Abelson murine leukemia viral oncogene homolog 1 gene; *MLL* mixed-lineage leukemia gene. ^a^Flow data from one patient was unavailable

Patient #1 achieved hematologic remission during cycle 1 of blinatumomab and complete MRD response by PCR (Supplementary Table 2), with MRD reappearance on day 29 by flow cytometry at a level of 0.01%. In cycle 3, day 29 of blinatumomab, approximately 3 months after achieving hematologic remission, patient experienced a hematologic relapse. Blasts at relapse were CD19− CD10+ CD22− CD34− CD38+ CD45dim CD58+ iCD79a+ (Fig. 1a, Supplementary Table 1). Patient received antileukemic medication after relapse and was alive at time of withdrawn consent.

**Fig. 1:**
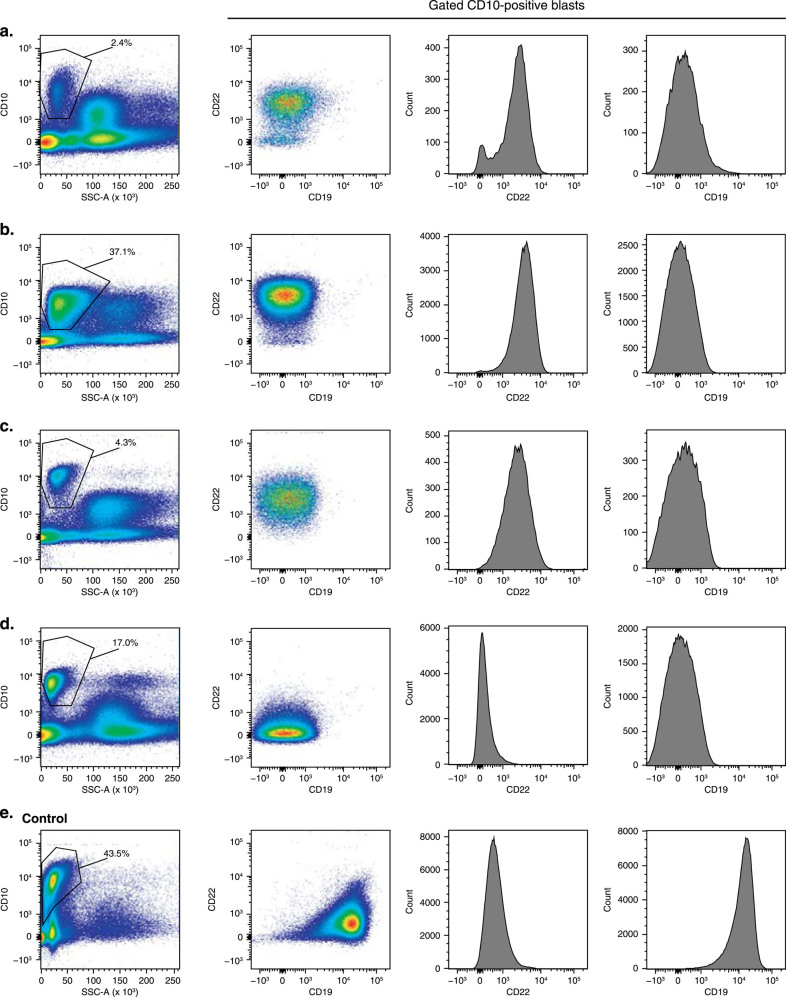
**Flow cytometric profiles of blasts from four patients with CD19-negative relapse after blinatumomab treatment and one control patient during treatment.** **a** Patient #1, **b** Patient #2, **c** Patient #3, **d** Patient #4, and **e** control patient

Patient #2 experienced two hematologic relapses on study (one CD19-positive and one CD19-negative). Patient achieved hematologic remission during cycle 1 of blinatumomab and complete MRD response by flow cytometry but was MRD-positive by PCR. In cycle 1, day 29, approximately 2 weeks after blinatumomab-induced hematologic remission, patient relapsed with primarily CD19-positive leukemic blasts, which included a small proportion of CD19− and CD10+ blasts (approximately 5%). Patient continued to receive blinatumomab and achieved a second hematologic remission in cycle 2, day 15, with an MRD-positive response by PCR (Supplementary Table 2) and MRD relapse by flow cytometry. Patient relapsed on day 29 with leukemic blasts that were CD19− CD10+ CD22+ CD34+ CD38+ CD45dim/− CD58+ CD66c− (8%+) (Fig. 1b, Supplementary Table 1). Patient received antileukemic medication after relapse and subsequently died due to disease progression before study completion.

Patient #3 achieved hematologic remission during cycle 1 of blinatumomab and complete MRD response by PCR (Supplementary Table 2) but MRD-positive response on CD19-negative blasts by flow cytometry. In cycle 3, day 29 of blinatumomab, approximately 3 months after blinatumomab-induced hematologic remission, patient relapsed with blasts that were CD19− CD10+ CD22+ CD33− CD34− CD38+ CD45dim CD58+ CD66c− (5%+) iCD79a+/− CD81+ (Fig. 1c, Supplementary Table 1). Patient received antileukemic medication after relapse and subsequently died due to disease progression before study end.

Patient #4 went into hematologic remission during cycle 1 of blinatumomab, with a complete MRD response by flow cytometry and PCR (Supplementary Table 2). During cycle 4 of blinatumomab, approximately 4.5 months after hematologic remission, patient relapsed on day 29 with blasts that were CD19− CD10+ CD22+ CD34+ CD38+ CD45dim/− CD58+ CD66c+ CD72+ iCD79a− CD81+ (Fig. 1d, Supplementary Table 1). Patient received antileukemic medication after relapse and was alive at the end of study.

Patient #5 had blasts that were CD19+ CD10− CD22+ CD34+/− iCD79a+ at study entry but converted from CD19-positive to CD19-negative after 10 days in cycle 1 of blinatumomab. Upon progression, patient had blasts that were CD2+ CD9+ CD11b+ CD11c+ CD38+ CD56+ CD64+, suggesting gain of a monocytic phenotype.

The flow cytometric profile for a control patient is shown for reference (Fig. 1e).

We found that CD22-positive blasts were present in three of four patients with CD19-negative relapse, suggesting that CD22 may be a helpful marker to monitor MRD, although CD22 expression on B cells has a broad and dim intensity distribution that can be lower than CD19 expression^10^. In total, four patients were identified primarily based on very bright CD10 expression and low side scatter. Other potential markers identified from our analyses included CD34 (two of four) and iCD79a (two of three). In three patients, CD45 was dim to negative, which, together with bright CD10 expression, was highly suggestive of CD19-negative relapse. CD66c was used as an aberrant marker for MRD monitoring in three cases, but this marker identified leukemic blasts in only one case. Although CD81 is known to form a complex with CD19/CD21 and was detected on blasts of the two patients analyzed, CD81 expression can be lost in cases of CD19-negative relapse^11, 12^, raising concerns about the utility of CD81 as a marker to identify ALL progression after CD19-negative relapse. CD72, another potentially useful B-cell-specific marker, was analyzed and detected in one patient (Patient #4).

CD19-negative relapse in B-precursor ALL is a significant problem, complicating accurate monitoring of disease progression, particularly during treatment with CD19-directed immunotherapies. In our study cohort, 22% (4/18) of patients with evaluable data were observed to have CD19-negative relapse. Two proposed mechanisms of CD19-negative relapse have been described to date: loss of the CD19 epitope and lineage switch^3, 4, 13^. Loss of the CD19 epitope has occurred after CD19-directed immunotherapy, such as CAR T-cell therapy, through deletions within *CD19*, de novo frameshift and missense mutations in exon 2 of CD19, or alternative splicing of CD19 mRNA^3^. We could not perform these molecular analyses within the reported clinical trial due to inadequate RNA availability. CD19-negative relapse can also occur through lineage switch of B-precursor cells from the lymphoid lineage to a CD14-positive myeloid lineage, a phenomenon reported to occur in 4% of B-precursor ALL^4, 13^. The four presented cases of patients who had CD19-negative relapse were unrelated to lineage switch, although lineage switch has been reported in a patient who relapsed during blinatumomab treatment^2^. There were three patients in this study with Philadelphia chromosome–positive ALL, but none had CD19-negative relapse^14^.

In this trial, MRD was evaluated in a portion of patients by PCR in addition to flow cytometry. MRD detected by PCR provides reliable results independently from CD19 expression. However, it does not identify this important change of CD19-negative relapse, which may have therapeutic implications. Loss of the CD19 antigen is also an important cause of discrepancy between flow cytometry and PCR. The ability to accurately monitor ALL progression during treatment and after CD19-negative relapse by flow cytometry requires identifying additional B-lineage or other specific markers consistently expressed on B cells. CD22 or CD24 in combination with markers abnormally expressed in B-precursor ALL (CD10, CD20, CD34, CD38, and CD45) have been shown to identify a subset of blasts in patients with CD19-negative relapse^15^.

Here, we identified CD22, iCD79a, CD72, and CD81 as potential alternatives or additional markers to aid in monitoring B-precursor ALL after CD19-negative relapse. However, because none of these markers was expressed consistently in all five patients, further studies in a larger cohort of patients are needed to confirm their specificity and reliability and to identify a more refined set of markers predictive of leukemic cell growth. As CD19-directed therapies are being more commonly used, identifying factors to predict CD19-negative relapse and monitor patients with CD19-negative relapse will be important.

## Electronic supplementary material

Supplementary Information (clean)
