# Supplementary material for: CD19-negative relapse of pediatric B-cell precursor acute lymphoblastic leukemia following blinatumomab treatment
Source: Blood Cancer J. 2017 Dec 20;7(12):659. doi: 10.1038/s41408-017-0023-x (PMC5802535; doi:10.1038/s41408-017-0023-x)
Supplement: Supplementary file 1 — Supplementary Information (clean) [file 41408_2017_23_MOESM1_ESM.doc]

**Supplementary Information**

**CD19-negative relapse of pediatric B-cell precursor acute lymphoblastic leukemia following blinatumomab treatment**

*Ester Mejstríková, Ondrej Hrusak, Michael J Borowitz, James A Whitlock, Benoit Brethon, Tanya M Trippett, Gerhard Zugmaier, Lia Gore, Arend von Stackelberg, Franco Locatelli*

Supplementary Methods

Supplementary Tables S1, S2

**Supplementary Methods**

**Study summary**

Detailed descriptions of study design, patient eligibility, dose modifications, interruptions, and discontinuation were previously reported.^1^ Briefly, this was an open‑label, single-arm, multicenter study from 26 European and United States centers enrolling pediatric and adolescent patients aged < 18 years with B-cell precursor acute lymphoblastic leukemia (ALL) and > 25% bone marrow blasts. Patients had disease that was primary refractory, in first relapse after full salvage induction regimen, in second or later relapse, or in any relapse after allogeneic hematopoietic stem cell transplantation. Blinatumomab was dosed by continuous intravenous infusion (4 weeks on/2 weeks off) for up to five cycles. During phase 1 of the study, the maximum tolerated dose was determined to be 15 µg/m^2^/day, and the recommended stepwise dosage (5 µg/m^2^/day for the first 7 days and 15 µg/m^2^/day thereafter) was subsequently used in the phase 2 portion of the study. Patients were assessed for remission status and minimal residual disease (MRD) determination at study entry, on days 15 and 29 of cycle 1, and on day 29 of additional cycles.

**Remission status**

Hematologic remission status was defined according to published criteria^2^ and classified as complete remission, hypocellular or acellular bone marrow, partial remission, stable disease, progressive disease, and hematologic relapse. Complete remission was defined as having no evidence of circulating blasts or extramedullary disease and < 5% blasts in an evaluable bone marrow. Partial remission was defined as a complete disappearance of circulating blasts, 5%–25% blasts in an evaluable bone marrow, and appearance of normal progenitor cells. The patient was considered to have stable disease if he/she did not meet the criteria of either complete or partial remission or progressive disease. Progressive disease was defined as 1) the greater of either a ≥ 25% increase or an absolute increase of ≥ 5,000 cells/µL in circulating leukemia cells, 2) development of extramedullary disease, or 3) other laboratory or clinical evidence of progressive disease. Presence of bone marrow leukemic blasts was considered as a hematologic relapse.

***Flow cytometry***

Flow cytometry methodology details have been previously published.^3^ Briefly, flow cytometry samples were processed within 48 hours of collection. Sample preparation consisted of a 15-minute incubation with monoclonal antibodies (mAbs) at the recommended sample-to-mAb volume ratios. Red blood cells were lysed in ammonium chloride followed by centrifugation and immediate data acquisition. For intracellular staining, the FIX & PERM® kit (An Der Grub Bioresearch, Kaumberg, Austria) was used. All events were acquired and stored in listmode files, and no live gate strategy was used. At diagnosis, a minimum of 20,000 events per tube were acquired. During MRD assessment, at least 300,000 cells were acquired.

***MRD by flow cytometry***

For patients #1 to #4, MRD by flow cytometry was assessed using a total of three mAb combinations: **1)** CD10 PE (clone SS2/36, Dako, purchased from MEDESA, catalog #R084801), CD19 PC7 (clone J4.119, Immunotech, catalog #IM3628), CD20 FITC (clone L27, BD, catalog #345792), CD34 APC (clone 581, Immunotech, catalog #IM2472), CD38 Ax700 (clone HIT2, EXBIO, catalog #A7-366-T100), CD45 PerCP (clone 2D1, BD, catalog #345809), and SYTO-41 (fluorescent dye–staining cells containing DNA/RNA for exclusion of debris from the analysis, Thermo Fisher Scientific, purchased from Life Technologies Czech Republic, catalog #S11352); **2)** CD58 FITC (clone AICD58, Immunotech, catalog #IM1218), CD markers according to the diagnostic phenotype in PE format, CD10 ECD (clone ALB 1, Immunotech, catalog #IM3608U), CD19 PC7 (clone J4.119 Immunotech, catalog #IM3628), CD20 PB (clone LT20, Exbio, catalog #PB-414-T100), CD34 APC (clone LIQ, Immunotech, catalog #IM2472), CD38Ax700 (clone HIT2, EXBIO, catalog #A7-235-T100 and catalog #A7-366-T100), and CD45 PerCP (clone 2D1, BD, catalog #345809) and **3)** SYTO-16 (fluorescent dye–staining cells containing DNA/RNA for excluding debris from the analysis, Thermo Fisher Scientific, purchased from Life Technologies Czech Republic, catalog #S-7578), CD19 PE (clone SJ25C, BD, catalog #345789), CD45 PerCP (clone 2D1, BD, catalog #345809), CD71Ax700 (clone MEM-75, Exbio, catalog #A7-235-T100), and DAPI (4',6-diamidin-2-fenylindol, cell permeable, fluorescent dye that binds to DNA was used for eventual assessment of sample viability, especially for long transport, KRD, catalog #D-3571). At study entry, all samples from patients #1 to #4 were assessed with the following markers: CD7 ECD (clone 8H8.1, Immunotech, catalog #A70202), CD10 PE (clone SS2/36, Dako, purchased from MEDESA, catalog #R084801), CD11c PercpCy5.5 (clone B-Ly6, BD, catalog #646784), CD13 PE (clone SJ1D1, Immunotech, catalog #A07762), CD14 APC (clone MφP9, BD, catalog #345787), CD15 FITC (clone MMA, BD, catalog #332778), CD19 PC7 (clone J4.119 Immunotech, catalog #IM3628), CD20 FITC (clone L27, BD, catalog #345792), CD22 FITC (clone SJ10.1H11, Immunotech, catalog #IM0779U), CD24 PE (clone ALB9, Immunotech, catalog #IM1428U), CD33 APC (clone D3HL60.251, Immunotech, catalog #IM2471), CD34 APC (clone 581, Immunotech, catalog #IM2472), CD38 Ax700 (clone HIT2, EXBIO, catalog #A7-366-T100), CD45 PerCp (clone 2D1, BD, catalog #345809), CD52 PE (clone YTH34.5, Serotec, purchased from Exbio, catalog #SFL1642PE serotec), CD58 FITC (clone AICD58, Immunotech, catalog #IM1218), CD66c PE (clone KOR-SA3544, Immunotech, catalog #IM2357U), CD79a FITC (clone ZL7-4, Serotec, purchased from Exbio, catalog #SEROTEC MCA 1298F), CD117 PE (clone 95C3, Immunotech, catalog #IM1360U), intracellular [i]CD79 PE (clone HM57, DAKO, purchased from MEDESA, catalog # R715901), iIgM (clone G20-127, BD, catalog #555782, iLysozyme PE (clone LZ-2, Nordic MUbioNordic MUbio, purchased from Exbio, catalog #GM-4133 Nordic Mubio), iMPO FITC (clone CLB-MPO-1, Immunotech, catalog #IM1874U), kappa FITC (polyclonal, Immunotech, catalog #A07706), lambda PE (polyclonal, Immunotech, catalog #A07706), and NG2 (clone 7.1, Immunotech, catalog #B92429). Samples from patient #5 were assessed with the following markers: CD2, CD9, CD10, CD11b, CD11c, CD13, CD19, CD20, CD22, CD33, CD34, CD38, CD45, CD56, CD58, CD64, CD66c, CD72, iCD79a, and CD81. MRD was classified as MRD positive (MRD ≥ 10^−4^ [no MRD relapse]), MRD response (MRD < 10^−4^), complete MRD response (no detectable blasts), MRD reappearance (MRD detectable after complete MRD response with no MRD relapse), and MRD relapse (increase of MRD level by at least one log).

***MRD by polymerase chain reaction (PCR)***

MRD was assessed by PCR for clonal immunoglobulin/T-cell receptor rearrangements for European patients as described previously.^4-7^ Two to three of the markers for MRD quantification were applied for each respective patient using the guidelines of the EuroMRD consortium.^8^ MRD by PCR was classified as defined above for flow cytometry (ie, MRD positive, MRD response, complete MRD response, and MRD relapse).

**References**

1. von Stackelberg A, Locatelli F, Zugmaier G, Handgretinger R, Trippett TM, Rizzari C *et al*. Phase I/phase II study of blinatumomab in pediatric patients with relapsed/refractory acute lymphoblastic leukemia. *J Clin Oncol* 2016; **34**: 4381–4389.

2. Lauten M, Möricke A, Beier R. Prediction of outcome by early bone marrow response in childhood acute lymphoblastic leukemia treated in the ALL-BFM 95 trial: differential effects in precursor B-cell and T-cell leukemia. *Haematologica* 2012; **97**: 1048–1056.

3. Mejstríková E, Fronková E, Kalina T, Omelka M, Batinić D, Dubravcić K *et al*. Detection of residual B precursor lymphoblastic leukemia by uniform gating flow cytometry. *Pediatr Blood Cancer* 2010; **54**: 62–70.

4. Langerak AW, Szczepański T, van der Burg M, Wolvers-Tettero IL, van Dongen JJ. Heteroduplex PCR analysis of rearranged T cell receptor genes for clonality assessment in suspect T cell proliferations. *Leukemia* 1997; **11**: 2192–2199.

5. Pongers-Willemse MJ, Seriu T, Stolz F, d’Aniello E, Gameiro P, Pisa P *et al*. Primers and protocols for standardized detection of minimal residual disease in acute lymphoblastic leukemia using immunoglobulin and T cell receptor gene rearrangements and TAL1 deletions as PCR targets: report of the BIOMED-1 CONCERTED ACTION - Investigation of minimal residual disease in acute leukemia. *Leukemia* 1999; **13**: 110–118.

6. Szczepanski T, Pongers-Willemse MJ, Langerak AW, Harts WA, Wijkhuijs AJ, van Wering ER *et al*. Ig heavy chain gene rearrangements in T-cell acute lymphoblastic leukemia exhibit predominant DH6-19 and DH7-27 gene usage, can result in complete V-D-J rearrangements, and are rare in T-cell receptor alpha beta lineage. *Blood* 1999; **93**: 4079–4085.

7. Szczepanski T, van der Velden VH, Hoogeveen PG, de Bie M, Jacobs DC, van Wering ER *et al*. Vdelta2-Jalpha rearrangements are frequent in precursor-B-acute lymphoblastic leukemia but rare in normal lymphoid cells. *Blood* 2004; **103**: 3798–3804.

8. van der Velden VH, Cazzaniga G, Schrauder A, Hancock J, Bader P, Panzer-Grumayer ER *et al*. Analysis of minimal residual disease by Ig/TCR gene rearrangements: guidelines for interpretation of real-time quantitative PCR data. *Leukemia* 2007; **21**: 604–611.

**SUPPLEMENTARY TABLES**

**Table S1**. Flow cytometric profiles of patients at study entry and at relapse

|  | **Patient #1** | | **Patient #2** | | **Patient #3** | | **Patient #4** | | **Patient #5** | |
| --- | --- | --- | --- | --- | --- | --- | --- | --- | --- | --- |
| **Marker** | **SE** | **Rel** | **SE** | **Rel** | **SE** | **Rel** | **SE** | **Rel** | **SE** | **Rel** |
| CD2 | ND | ND | ND | ND | ND | ND | ND | ND | ND | + |
| CD3 | ND | − | ND | − | ND | − | ND | − | ND | ND |
| CD4 | ND | − | ND | − | ND | − | ND | − | ND | ND |
| CD5 | − | ND | − | ND | − | ND | ND | ND | ND | ND |
| CD7 | − | ND | − | ND | ND | ND | ND | ND | ND | ND |
| CD8 | ND | − | ND | − | ND | − | ND | − | ND | ND |
| CD9 | ND | ND | ND | ND | ND | ND | ND | + | + | + |
| CD10 | + | + | + | + | + | + | + | + | − | ND |
| CD11b | ND | ND | ND | ND | ND | ND | ND | ND | ND | + |
| CD11c | − | ND | − | ND | − | ND | − | ND | ND | + |
| CD13 | − | ND | − | ND | − | ND | − | ND | − | ND |
| CD14 | − | − | − | − | ND | − | − | − | ND | ND |
| CD15 | − | ND | − | ND | − | ND | − | ND | ND | ND |
| CD19 | + | − | + | − | + | +/− | + | − | + | − |
| CD20 | − | − | − | −^a^ | + | +/−^b^ | − | − | − | ND |
| CD22 | + | − | +/− | + | + | + | + | + | + | ND |
| CD24 | + | ND | + | ND | + | ND | + | ND | ND | ND |
| CD27 | ND | ND | ND | ND | ND | ND | ND | − | ND | ND |
| CD33 | − | ND | − | ND | +/− | − | − | ND | − | ND |
| CD34 | − | − | + | + | − | − | +/− | + | +/− | ND |
| CD38 | + | + | + | + | + | + | + | + | + | + |
| CD44 | ND | ND | ND | ND | ND | ND | ND | +/− | ND | ND |
| CD45 | dim/− | dim | dim/− | dim/− | dim | dim | dim/− | dim/− | + | ND |
| CD49f | ND | ND | ND | ND | ND | ND | ND | dim/− | ND | ND |
| CD52 | − | ND | +/− | ND | + | ND | + | ND | ND | ND |
| CD56 | ND | ND | ND | ND | ND | ND | ND | ND | ND | + |
| CD58 | + | + | + | + | + | + | + | + | + | ND |
| CD64 | ND | ND | ND | ND | ND | ND | ND | ND | ND | + |
| CD66c | − | ND | +/− | −s | − | −s | +/−^b^ | + | ND | ND |
| CD66c-CD123 | ND | ND | ND | ND | ND | ND | ND | + | ND | ND |
| CD71 | ND | − | ND | − | ND | − | ND | − | ND | ND |
| CD72 | ND | ND | ND | ND | ND | ND | ND | + | ND | ND |
| CD81 | ND | ND | ND | ND | ND | + | ND | + | ND | ND |
| CD117 | − | ND | − | ND | − | ND | − | ND | ND | ND |
| CD123 | ND | ND | ND | ND | ND | ND | ND | − | ND | ND |
| CD200 | ND | ND | ND | ND | ND | ND | ND | + | ND | ND |
| HLA DR | ND | + | ND | + | ND | + | ND | − | ND | ND |
| Kappa chain | − | ND | − | ND | − | ND | − | ND | ND | ND |
| Lambda chain | − | ND | − | ND | − | ND | − | ND | ND | ND |
| NG2 | − | ND | − | ND | − | ND | − | ND | ND | ND |
| iCD3 | − | ND | − | ND | − | − | − | − | ND | ND |
| iCD79a | + | + | + | ND | + | +/− | + | − | + | ND |
| iIgM | − | ND | − | ND | − | ND | − | ND | ND | ND |
| iLysozyme | − | ND | − | ND | − | ND | − | ND | ND | ND |
| iMPO | − | ND | − | ND | − | − | − | − | ND | ND |
| iTdT | ND | ND | ND | ND | ND | ND | ND | + | ND | ND |

Abbreviations: HLA DR, human leukocyte antigen-antigen D related; i, intracellular; IgM, immunoglobulin M; MPO, myeloperoxidase; ND, not determined; NG2, neuron-glial antigen 2; Rel, at relapse; SE, study entry; TdT, terminal deoxynucleotidyl transferase; −s, negative with small subset (5%–8%) positive. ^a^Subclone 12%+. ^b^Subclone 35%+.

**Table S2**. MRD analysis by PCR of Ig/TCR clonal rearrangements for four patients who went into remission after blinatumomab treatment

| **Patient** | **Marker** | **Quantitative Range** | **Sensitivity** | **Present at Remission** | **Present at Relapse** |
| --- | --- | --- | --- | --- | --- |
| Patient #1 | Kintron-kde | 1x10^−4^ | 1x10^−5^ | No | Yes |
|  | VH3DH6JH4 | 1x10^−4^ | 1x10^−5^ | No | Yes |
|  | Vγ1JP1/2 | 5x10^−4^ | 1x10^−5^ | No | Yes |
| Patient #2 | VH3DH7JH4 | 5x10^−4^ | 1x10^−5^ | Yes | Yes |
|  | VH6DH4JH1 | 5x10^−4^ | 1x10^−5^ | Yes | Yes |
|  | Vδ2Dδ3Jα29 | 1x10^−4^ | 1x10^−5^ | Yes | Yes |
| Patient #3 | DH6JH4 | 1x10^−3^ | 5x10^−4^ | No | Yes |
|  | VH7DH6JH4 | 5x10^−4^ | 1x10^−4^ | No | Yes |
| Patient #4 | Vd2Dd3 | 1x10^−4^ | 1x10^−5^ | No | Yes |
|  | VH2JH4 | 1x10^−3^ | 5x10^−4^ | No | Yes |
|  | VH3JH5 | 5x10^−4^ | 1x10^−4^ | No | Yes |

Abbreviations: Ig, immunoglobulin; MRD, minimal residual disease; ND, not determined; PCR, polymerase chain reaction; TCR, T‑cell receptor.
